# *Dermestes maculatus*: an intermediate-germ beetle model system for evo-devo

**DOI:** 10.1186/s13227-015-0028-0

**Published:** 2015-10-16

**Authors:** Jie Xiang, Iain S. Forrest, Leslie Pick

**Affiliations:** Department of Entomology, University of Maryland, 4112 Plant Sciences Building, College Park, MD 20742 USA; Program in Molecular and Cell Biology, University of Maryland, 4112 Plant Sciences Building, College Park, MD 20742 USA

**Keywords:** Evo-devo, Segmentation, Pair-rule patterning, *paired*, RNAi, *Dermestes maculatus*

## Abstract

**Background:**

Understanding how genes change during evolution to direct the development of diverse body plans is a major goal of the evo-devo field. Achieving this will require the establishment of new model systems that represent key points in phylogeny. These new model systems must be amenable to laboratory culture, and molecular and functional approaches should be feasible. To date, studies of insects have been best represented by the model system *Drosophila melanogaster*. Given the enormous diversity represented by insect taxa, comparative studies within this clade will provide a wealth of information about the evolutionary potential and trajectories of alternative developmental strategies.

**Results:**

Here we established the beetle *Dermestes maculatus*, a member of the speciose clade Coleoptera, as a new insect model system. We have maintained a continuously breeding culture in the lab and documented *Dermestes maculatus* embryogenesis using nuclear and phalloidin staining. Anterior segments are specified during the blastoderm stage before gastrulation, and posterior segments are added sequentially during germ band elongation. We isolated and studied the expression and function of the pair-rule segmentation gene *paired* in *Dermestes maculatus*. In this species, *paired* is expressed in stripes during both blastoderm and germ band stages: four primary stripes arise prior to gastrulation, confirming an intermediate-germ mode of development for this species. As in other insects, these primary stripes then split into secondary stripes. To study gene function, we established both embryonic and parental RNAi. Knockdown of *Dmac*-*paired* with either method resulted in pair-rule-like segmentation defects, including loss of Engrailed expression in alternate stripes.

**Conclusions:**

These studies establish basic approaches necessary to use *Dermestes maculatus* as a model system. Methods are now available for use of this intermediate-germ insect for future studies of the evolution of regulatory networks controlling insect segmentation, as well as of other processes in development and homeostasis. Consistent with the role of *paired* in long-germ *Drosophila* and shorter-germ *Tribolium*, *paired* functions as a pair-rule segmentation gene in *Dermestes maculatus.* Thus, *paired* retains pair-rule function in insects with different modes of segment addition.

**Electronic supplementary material:**

The online version of this article (doi:10.1186/s13227-015-0028-0) contains supplementary material, which is available to authorized users.

## Background

Understanding the basis for the diversity of plant and animal systems on our planet will require studies of the mechanistic basis of body patterning and developmental strategies used in different species as well as an understanding of how these mechanisms evolved (evo-devo). It is crucial that these studies include sampling of species from a broad range of taxa that represent distinct branches of the tree of life (reviewed in [1]). Rapid progress in the development of genomic technologies has made it possible to readily identify genes in diverse species. However, understanding how these genes control developmental processes will require establishment of model systems in which gene function can be assessed.

Arthropods represent ~80 % of all described species; among them, insects are the dominant taxa, representing ~65 % of all animal species on the planet [2]. Insects are easy to experimentally manipulate, can often be readily cultured in the laboratory, producing large numbers of embryos with reasonable generation time, and their enormous diversity makes them an ideal group for comparative studies to probe phenotypic diversity and unravel ancestral mechanisms. Among insects, the most sophisticated model system available to date is *Drosophila melanogaster* (*D. melanogaster*). *D. melanogaster* serves as a reference species for any study of insects, or other new animal model, with more than 100 years of study by thousands of researchers throughout the world, a plethora of genetic tools to assess gene function, and progress on every type of ‘omics’ analysis [3]. *D. melanogaster* is a member of the group of holometabolous insects thought to have arisen 300–400 million years ago (Mya) [4], which includes >80 % of all extant insect species [5]. Additional models are needed from this group to understand diversity in Holometabola. The most speciose order of holometabolous insects is Coleoptera (beetles), with >350,000 named species representing ~40 % of all insect species [2, 6, 7]. Coleoptera are thought to have arisen ~285 Mya [8] and have radiated to occupy a broad variety of niches on our planet including those with extreme environments, such as the Arctic, high mountain altitudes and dry, desert terrains. Beetles range in size from <0.5 mm to >15 cm in length and feed on everything from other insects, to fungus, decaying wood, a wide variety of plants, animal debris and even dung. The most sophisticated coleopteran model system developed to date is the flour beetle, *Tribolium castaneum* (*T. castaneum*; [9–11]), providing a frame of reference for the development of additional beetle systems to represent the diversity of this large clade.

Segmentation is a highly conserved feature shared by arthropods and outgroups [12–16]. Despite this similarity, the ways in which segments form and the genes that control this process vary among taxa [17–19]. Krause first classified insect embryogenesis into short-, intermediate- and long-germ modes based on the relative size of the germ anlage prior to gastrulation [20]. These different modes of segmentation can be distinguished by the number of segments established in the germ rudiment before gastrulation: long-germ (all or most segments are established more or less simultaneously), short-germ (only anterior segments are specified) and intermediate-germ (head and thorax segments, and sometimes anterior abdominal segments, are specified). Both short- and intermediate-germ insects differ from long-germ insects in that posterior segments are added sequentially from a posterior segment addition zone (SAZ) or growth zone. This strategy of ‘sequential addition’ of segments is thought to be ancestral to arthropods and it is only in holometabolous insects that long-germ development has been observed [21]. Phylogenetic studies and accumulating molecular evidence indicate that long-germ development in different orders of Holometabola has evolved independently [17, 22]. How modes of segment formation switched without disrupting the segmented body plan itself is unclear. The presence of nurse cells, enlarged germ size, acquisition of an anterior patterning center, shifted gap gene expression boundaries, and diminished activity of a segmentation clock have been proposed as prerequisites for long-germ development [17–19, 22–24]. Studies of the mechanisms underlying segmentation in an intermediate-germ insect, which may reflect an intermediate state between short- and long-germ modes of segmentation, will yield information on the transition from ancestral sequential specification to long-germ development. In addition, since long-germ development appears to have evolved several times independently within Holometabola, it will be of interest to compare mechanisms in species within a single clade rather than just comparing all sequentially segmenting species to *D. melanogaster*. These comparative studies will distinguish stages in the evolution of the long-germ mode which may have been gradual, with increasing numbers of segments specified simultaneously in different species, or may have occurred in a punctuated fashion, reflecting developmental constraints that remain to be discovered.

The two best-developed insect systems, *D. melanogaster* and *T. castaneum*, represent different modes of segment addition with *D. melanogaster* displaying the long-germ mode and *T. castaneum* specifying segments sequentially. Genetic screens in *D. melanogaster* identified a group of pair-rule segmentation genes (PRGs) that control the formation of body segments, and many of these also function in segmentation in *T. castaneum* [25–27]. However, their specific roles in the segmentation process often differ and some genes involved in segmentation in *D. melanogaster* do not function in segmentation in *T. castaneum* [25, 28]. Work from other insects suggests that new genes may be recruited into PRG networks and that PRG orthologs have acquired novel function in different lineages [29, 30]. To understand the extent to which mechanisms regulating segmentation vary, the genetic underpinnings of this process must be examined in different species. As first pointed out by Patel and Davis, Coleoptera are an ideal order for this comparison, as short-, intermediate- and long-germ development have all been observed in beetles [17, 31, 32]. Comparison of gene function in species within the same clade displaying these different developmental strategies will provide information about the extent of variation among segmentation regulatory networks, the impact of these changes on downstream targets, and clues about how changes in gene expression and function drive the evolution of alternative developmental modes.

Here we have established *Dermestes maculatus* (*D. maculatus*) as a system for comparative studies within Coleoptera. *T. castaneum* and *D. maculatus* diverged close to the time of origin of this clade ~250 Mya, [33], making this pair of species ideal for comparative studies, as they represent divergent lineages within the order Coleoptera. *D. maculatus* display an intermediate-germ mode of segmentation compared to the shorter-germ mode of *T. castaneum*. *D. maculatus* are easy to rear in the lab, with high fecundity and a short life cycle. We characterized the early steps of nuclear division in *D. maculatus* embryos and isolated an ortholog of the *D. melanogaster* PRG, *paired (prd). Dmac*-*prd* has pair-rule-like expression and function, regulating the expression of alternate stripes of the segment polarity gene *engrailed* (*en*). These studies support the conclusion that the function of *prd* as a PRG is highly conserved across holometabolous taxa. Additionally, these studies establish methods for in situ hybridization, antibody staining, and both parental and embryonic RNAi in *D. maculatus*.

## Methods

### *Dermestes* species verification using DNA barcoding

*D. maculatus* adults and larvae were purchased from Carolina Biological Supply Company. To verify the identity of the species, we amplified the mitochondrial cytochrome c oxidase subunit I (COI) gene [34, 35]. Genomic DNA was extracted using a DNeasy Tissue kit (Qiagen). Only wings and legs were taken from four *Dermestes* adults to avoid contamination by gut content. PCR using the primer pair LCO1490 (5′-GGTCAACAAATCATAAAGATATTGG-3′) and HCO2198 (5′-TAAACTTCAGGGTGACCAAAAAATCA-3′) amplified an approximately 700 base pair (bp) fragment. The sequence of this fragment matched *D. maculatus* COI (GenBank ID HM909035.1) except at position 581 (C to T transition, Additional file 1).

### Rearing of *D. maculatus*

*D. maculatus* were kept in large plastic cages (14.5 in. long × 8.5 in. wide × 10 in. high) with a thin layer of wood shavings spread on the bottom. The beetles were fed cat food (Fancy Feast) placed in a small weigh boat and changed twice a week. No water was added to avoid fungal growth. As immobile final instar larvae and pupae would be slaughtered by younger larvae, chunks of styrofoam were placed in the cages for the larvae to crawl into and hide before eclosion. Mesh cloth was used to cover the cages to prevent beetle escape while keeping the cages well ventilated. Cages were placed in incubators at 25 or 30 °C for colony maintenance. To collect embryos, newly eclosed *D. maculatus* were selected from the colony and placed in small plastic cages (9 inches long × 6 inches wide × 6.5 inches high) without wood shavings. They were fed daily to provide sufficient food. Cotton balls were stretched out and placed in the cage for egg laying. The cages were held at either 25 or 30 °C for developmental staging.

### Embryo collection and fixation

The protocol for fixation of *D. maculatus* embryos was modified from standard *D. melanogaster* and *Oncopeltus fasciatus* (*O. fasciatus*) embryo fixation protocols [36, 37] as follows. Cotton balls were carefully torn apart to let embryos fall onto a black sheet of paper. Embryos are white, approximately 0.2 cm in length, and can be seen easily against the black background. Embryos were transferred into small beakers and treated with 50 % bleach for 4 min followed by several water rinses. Embryos were then transferred into 1.5 ml Eppendorf tubes with distilled H_2_O (approximately 200 µl of embryos in 1000 µl of distilled H_2_O). Tubes were placed in boiling water for 3 min and then on ice for 7 min to swell the eggshell, making embryos easier to dissect before staining. Embryos were then fixed in heptane: 4 % PFA 1:1 for 20 min on a shaker at high speed (~250 rpm). PFA (lower phase) was removed and MeOH was added and the tube was shaken vigorously for 20 s. After several MeOH washes, embryos were stored at −20 °C in MeOH. A detailed *D. maculatus* embryo fixation protocol is provided (Additional file 2).

### *prd* gene cloning and identification

To isolate *prd* from *D. maculatus* embryonic mRNA, total RNA was extracted from 0 to 1 day (0–1 day) after egg laying (AEL) embryos developing at 30 °C using TRIzol (Invitrogen) and an RNeasy mini kit (Qiagen). Reverse transcription was performed using the QuantiTect Reverse Transcription kit (Qiagen) to prepare 0–1 day embryonic cDNA. Two rounds of degenerate PCR were performed (forward outer primer: *prd*-deg1 F: 5′-GGNGGNGTNTTYATHAAYGG-3′, GGVFING; reverse outer primer: *prd*-deg1 R: 5′-RTTNSWRAACCANACYTG-3′, QVWFSN; forward inner primer: *prd*-deg2 F: 5′-MARATHGTNGARATGGC-3′, KIVEMA; reverse inner primer: *prd*-deg2 R: 5′-RTANACRTCNGGRTAYTG-3′, QYPDIY; [38]), generating a product of approximately 600 bp length. After purification and insertion into pGEM-T Easy Vector (Promega) by TA cloning, sequencing of individual clones revealed partial *Dmac*-*prd*, as well as partial sequences of the *Pax3/7* family genes *Dmac*-*gooseberry (gsb)* and *Dmac*-*gooseberry*-*neuro* (*gsb*-*n*) [39–41]. The 3′ end of the *Dmac*-*prd* coding sequence and 3′ UTR were isolated through two rounds of 3′RACE using gene-specific primers and the FirstChoice RLM-RACE kit (Ambion) following the manufacturer’s instructions (1st round outer primer: AGAAACAGGCTCGATTCGTC, 1st round inner primer GATCGTCTCGTCAAGGAAGG; 2nd round outer primer: 5′ TTAGCTGGTGGCATTCAAAA, 2nd round inner primer 5′ AAGCTCTGTTGGTGCTGGTT). A contiguous fragment spanning part of the paired domain (PD) through the stop codon was verified using gene-specific primers: *Dmac*-*prd*3′F 5′ AGAAACAGGCTCGATTCGTC and *Dmac*-*prd*3′R 5′ CAGTTGGGTAACTCAGTGAACG. The region coding for the C-terminus of the PD through the stop codon was inserted into the XhoI and XbaI restriction sites of a KS vector for use as template for RNA in situ hybridization probe and double-stranded RNA (dsRNA) syntheses (KS-*Dmac*-*prd*).

### Embryo developmental staging, RNA in situ hybridization and antibody staining

For *D. maculatus* developmental staging, embryos were collected every 2 h (h) AEL over an 18-h period. After fixation, as described above, MeOH was removed and embryos were transferred into glass dishes with PBST. They were then hand-dissected with Dumont #5 forceps. For staging, embryos were incubated with 1:1000 SYTOX Green (Invitrogen) in the dark for 30 min at room temperature. They were then washed three times with PBST and visualized under fluorescence microscopy (Olympus SZX12, Leica 501007, or Leica SP5X). *D. melanogaster* protocols were followed for tracking the cytoskeletal dynamics using phalloidin and DAPI nuclear staining [42]. For phalloidin staining, 80 % EtOH was used instead of MeOH for fixation. After hand-dissection in PBTA (1× PBS, 0.1 % TritonX-100, 0.02 % sodium azide), embryos were incubated with Alexa Fluor 488 phalloidin (1:200; Molecular probes) overnight at 4 °C and then washed several times with PBST. Embryos were mounted in Vectashield mounting solution with DAPI (Vector Laboratories) and visualized with confocal microscopy (Leica SP5X). For in situ hybridization, digoxygenin-labeled *Dmac*-*prd* probes were synthesized using T7 polymerase (antisense) or T3 polymerase (sense) (Roche). The in situ hybridization was performed following modifications of a standard *D. melanogaster* RNA in situ hybridization protocol [43] (see Additional file 2 for details). Briefly, fixed embryos were hand-dissected in PBST. Embryos were pre-hybridized in hybridization solution for one h at 60 °C. After overnight incubation with 1:50 of digoxygenin-labeled probe (~10 ng/µl final concentration) at 60 °C, embryos were washed in hybridization solution and PBST. AP conjugated sheep anti-digoxygenin antibody (1:2000; Roche) was added. Embryos were incubated for one h at room temperature. Following four washes with PBST, NBT/BCIP (Roche) was used for detection. Antibody staining was performed following a standard *D. melanogaster* protocol [44, 45]. Hand-dissected fixed embryos were incubated with anti-En 4D9 primary antibody (1:5 dilution of antibody stock provided by the Developmental Studies Hybridoma Bank at 53 µg/ml) and then with biotinylated anti-mouse antibody (1:500; Vector Laboratories). A color reaction was performed after ABC (Vector Laboratories) incubation using DAB (Sigma). Embryos were incubated with SYTOX Green in PBST, washed three times in PBST, and visualized with Olympus SZX12, Leica 501007, or Zeiss SteREO Discovery. V12 microscopy. Embryos at germ band stages were hand-dissected to remove yolk before visualization.

### Parental and embryonic RNA interference and phenotypic analysis

Primers with T7 promoter sequence at their 5′ ends were used to amplify fragments from KS-*Dmac*-*prd.* (5′ region: STPYAP to VQPSSS, forward primer: *Dmac*-*prd*RNAi5′F: 5′ taatacgactcactatagggagaTTCAACTCCATACGCACCAA, reverse primer: *Dmac*-*prd*RNAi5′R: 5′ taatacgactcactatagggagaTGATGAACTCGGTTGCACAT; 3′ region: SANSNS to NPSKTF, forward primer: *Dmac*-*prd*RNAi3′F: 5′ taatacgactcactatagggagaAGTGCCAATAGCAACAGCAA, reverse primer: *Dmac*-*prd*RNAi3′R: 5′ taatacgactcactatagggagaCCGAAGGTTTTTGATGGATT). The PCR products were used as templates for dsRNA syntheses. MEGAscript T7 Transcription kit (Ambion) was used to make dsRNA according to the manufacturer’s instructions. For parental RNAi, pupae were selected from the *D. maculatus* colony. Female and male pupae were separated by visualizing their genitalia (Additional file 3). 2 µl of dsRNA (2 µg/µl) was injected into the abdomen of each newly eclosed female. After 1 day recovery at 30 °C, injected females were mated by placing them in small plastic cages with an equal number of uninjected males. After allowing them to mate for 1 day, cotton balls were added to cages and embryos were collected daily for phenotypic analysis. For embryonic RNAi, 0–3 h AEL embryos (pre-cellular blastoderm) were collected at 25 °C and aligned on glass slides. Approximately 50–100 ng (3 μg/μl) dsRNA was injected into each embryo using a micromanipulator within 5 h AEL. To examine morphological defects, hatched larvae were collected and fixed in #1184C Pampel’s solution (BioQuip Products, Inc.) at 4 °C overnight before visualization. To screen for segmentation defects, each larva was stretched out using forceps under a dissecting microscope. To examine Engrailed (En) expression, embryos at appropriate stages were fixed and stained, as described above.

## Results

### Early embryogenesis in *D. maculatus*

**Fig. 1:**
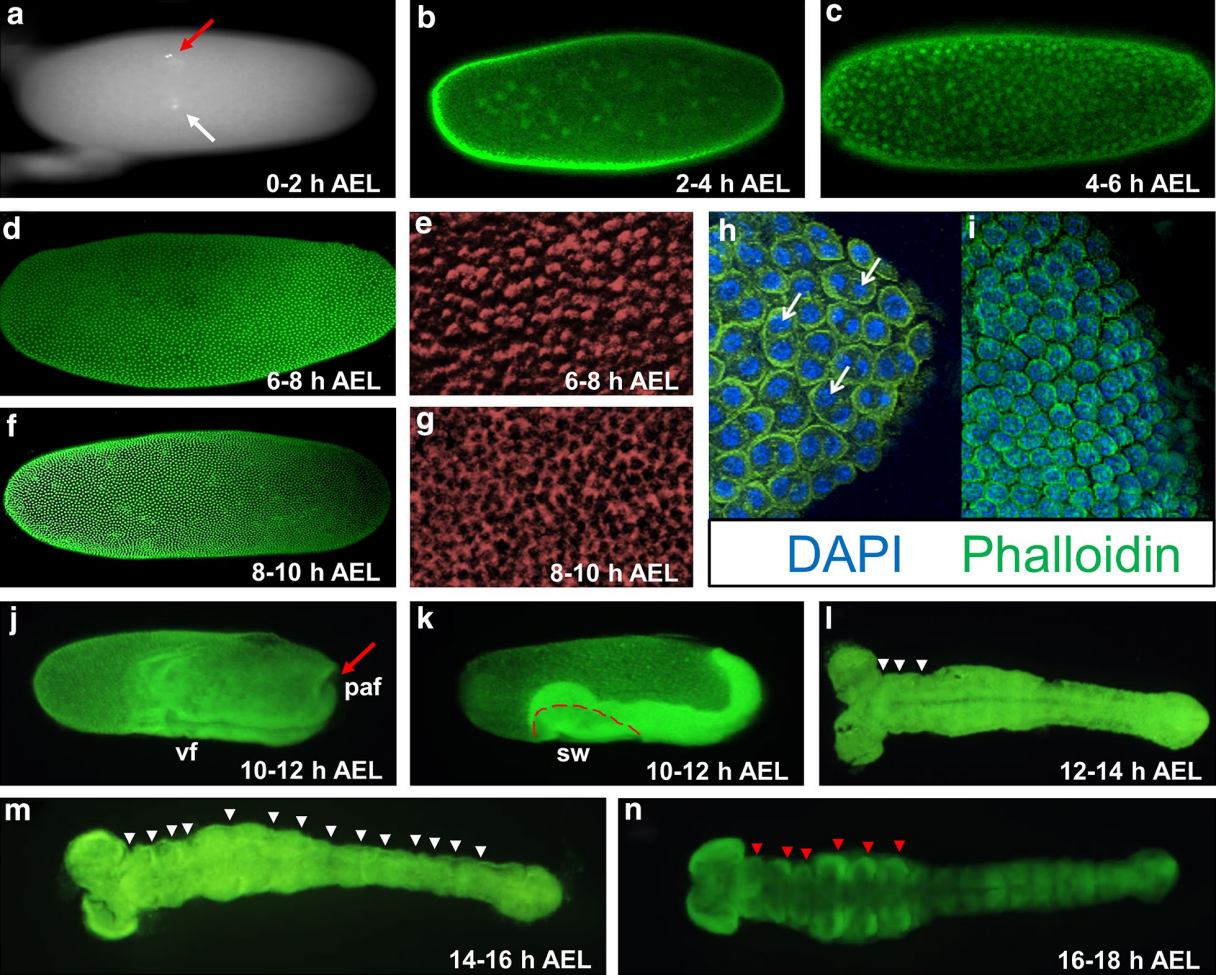
Early *D. maculatus* embryogenesis. Photographs of *D. maculatus* embryos are shown, documenting key steps of nuclear division and early embryonic development. **a** DAPI nuclear staining of a 0–2 h AEL *D. maculatus* embryo. **b**–**d**, **f**, **j**–**n** Nuclear staining using SYTOX Green of *D. maculatus* embryos between 2 and 18 h AEL, as indicated. **e**, **g** F-actin phalloidin staining of 6–8 h and 8–10 h AEL *D. maculatus* embryos (*recolored red*). **h**, **i** Merge of DAPI (*blue*) and phalloidin (*green*). **a** *White arrow* indicates pronuclei. *Red arrow* indicates polar body nuclei. **b** Nuclei have divided and spread in the central portion of the embryo. **c** Nuclei continue to divide and migrate towards the egg surface. **d** Most nuclei have arrived the periphery of the egg. **e** “Cap”-like phalloidin staining suggests the arrival of nuclei at the surface. **f** Cells have rearranged as some are closely clustered together in the ventral posterior area. **g** “Furrow canal”-like phalloidin staining appears during this stage. **h**, **i** Fully cellularized embryo. *White arrows* indicate cells at telophase of mitosis on the egg surface. **j** The ventral furrow (vf) has invaginated and posterior amniotic fold (paf, *red arrow*) has appeared. **k** The germ band has coalesced and begun to extend towards the dorsal side of the embryo. *Red dashed line* indicates serosal window (sw). **l** An extending germ band stage embryo with bilateral head lobes. *White arrowheads* show segmental furrows. **m** Segmental furrows appear in more posterior regions as the germ band elongates (*white arrowheads*). **n** A fully elongated germ band with morphological segments and appendage primordia (*red arrowheads* indicate appendage primordia). Embryos were reared at 25 °C and photographed with Olympus SZX12, Leica 501007 or Leica SP5X confocal microscopy

Since little was known about the early stages of *D. maculatus* embryonic development, we tracked nuclear and cytoskeletal dynamics using SYTOX Green, DAPI and phalloidin staining (Fig. 1). Progression of embryogenesis was monitored at 25 °C to slow development and capture all stages. Zygotic nuclei were first observed dividing multiple times in the center of the embryo, forming a syncitium (0–6 h AEL, Fig. 1a–c). At very early stages, female and male pronuclei were evident inside the embryo (white arrow, Fig. 1a), while the polar body nuclei were at the surface of the embryo (red arrow, Fig. 1a). After several divisions, zygotic nuclei gradually distributed along the length of the embryo (Fig. 1b) and, after additional divisions, began migrating toward the egg surface (Fig. 1c). Between 6 and 8 h AEL, most of the nuclei had migrated to the periphery of the egg, forming a syncytial blastoderm (Fig. 1d). “Cap”-like phalloidin staining was detected in some embryos at this stage, suggesting that nuclei arriving at the surface of the embryo are surrounded by cytoplasmic regions containing cytoskeleton (Fig. 1e). These phalloidin-stained actin caps protruded at the embryo surface, similar to cytoskeletal events that occur at a comparable stage in *D. melanogaster* (cell cycle 9/10; [46, 47]). Later, cell membranes formed between individual energids (nucleus with associated cytoplasm) as “furrow canal”-like phalloidin staining appeared, and a cellular blastoderm was established (8–10 h AEL, Fig. 1g). This is similar to cellularization events in *D. melanogaster* at cell cycle 14 [46, 47]. In *D. maculatus*, we were able to capture embryos in which dividing cells with two nuclei still sharing cytoplasm were visible at the cellular blastoderm surface (arrows, Fig. 1h), while cells that had finished cytokinesis each exhibited one nucleus enclosed by its own, individual membrane (Fig. 1i).

Between 10 and 12 h AEL, the *D. maculatus* embryo was rapidly transformed from a uniform cellular blastoderm to an elongating germ band (Fig. 1j, k, Additional file 4). In late cellular blastoderm, cells in the ventral posterior region packed together, forming the germ rudiment (Additional file 4a). The first detectable sign of gastrulation was the formation of a ventral furrow (vf), which appeared as a shallow broad furrow in the mid-ventral region (Additional file 4b, b’). Shortly after, several transverse folds emerged (Additional file 4b). As the ventral furrow further invaginated into the interior of the egg, it elongated towards the ventral posterior end (Additional file 4c’). The anterior-most fold embedded deeper while other short-lived transverse folds became invisible due to cell movements (Additional file 4c, c’). The dorsal embryonic region condensed while the dorsal anterior extraembryonic region expanded with gastrulation progression (compare Additional file 4b, c, arrowheads indicate the boundary between extraembryonic region and the embryo proper). Gastrulation proceeded as the ventral furrow became narrower and reached the posterior end (Fig. 1j; Additional file 4d, d’). Head lobes (hl) were visibly distinguished from surrounding extraembryonic tissue (Fig. 1j; Additional file 4d, d’). During the same time period, a posterior amniotic fold (paf) emerged and, shortly after, covered the posterior end of the germ anlage (red arrow in Fig. 1j; red arrowhead in Additional file 4d). It continued to proceed anteriorly along the ventral side as the germ band elongated (red arrowhead, Additional file 4e, e’). By approximately 12 h AEL, an early germ band with serosal window (sw) was established (Fig. 1k, red dashed line). The germ band further extended dorsally over the next 4 h and segmental furrows appeared in an anterior to posterior progression (12–16 h AEL; white arrowheads in Fig. 1l, m). Morphological segments as well as appendage primordia were seen at 16–18 h AEL (red arrowheads in Fig. 1n).

In sum, *D. maculatus* embryogenesis progressed through pre-blastoderm, cellular blastoderm, gastrulation and germ band extension stages within the first 18 h AEL at 25 °C. As expected, at 30 °C, embryos developed faster: a cellular blastoderm formed and gastrulation began between 4 and 6 h AEL. An early germ band was established 6–8 h AEL and the embryo reached late germ band stages within 10 h AEL (Additional file 5).

### Isolation of *prd* from *D. maculatus*

**Fig. 2:**
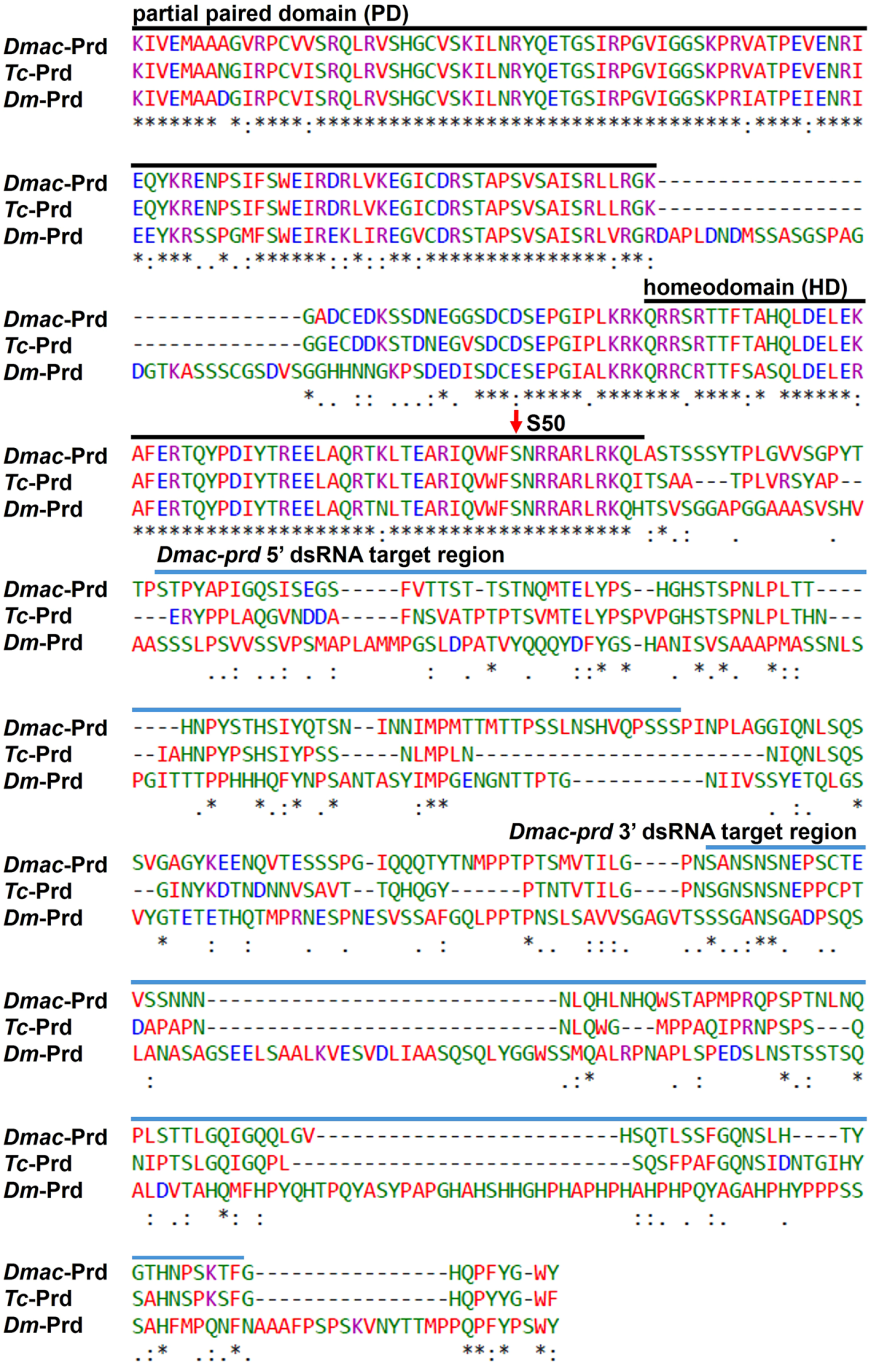
*Dmac*-Prd is similar to Prd from other insects. Alignment of partial Paired (Prd) sequences from *D. maculatus*, *T. castaneum*, and *D. melanogaster* is shown. *Black lines* indicate the paired domain (PD) and homeodomain (HD). *Red arrow* indicates S50 in the HD, critical for DNA-binding specificity. The regions used for RNAi experiments are overlined in *blue*. Protein sequence alignment was performed using ClustalW2. *Asterisk* indicates identical residues; *colon* indicates conserved substitutions; *full stop* indicates weakly similar substitutions. *Colors* indicate residues classified into groups according to their physicochemical properties. *Red* Nonpolar side chain; *Green* Polar side chain; *Blue* Negatively charged side chain; *Magenta* Positively charged side chain

To identify *Dmac*-*prd* ortholog(s), degenerate primers were designed based on conserved sequences in the paired domain (PD) and the homeodomain (HD) in Pax3/7 orthologs [38, 39, 48, 49]. An approximately 600 bp fragment isolated by PCR amplification using *Dmac* 0–1 day cDNA was extended by two rounds of 3′RACE to generate a 1341 bp fragment that encodes a PD and a HD (Fig. 2; *Dmac*-*prd* GenBank Accession number KT875123). An octapeptide sequence (OP) is present in most Pax3/7 orthologs but is absent from Prd from *D. melanogaster*, *T. castaneum*, *Apis mellifera* (*A. mellifera*) and *Nasonia vitripennis* (*N. vitripennis*) [50, 51]. This OP was not found in the *Dmac*-Prd sequence, consistent with this being an ortholog of *prd*, rather than another family member. The HD of this predicted *Dmac*-Prd has a serine residue at position 50 (red arrow, Fig. 2), which is vital for the DNA-binding specificity of Prd-family homeodomains [26, 38, 50, 52]. As shown in Fig. 2, the PD and the HD from *D. maculatus*, *T. castaneum* and *D. melanogaster* are similar. The PD of *Dmac*-Prd is 97 % identical to that of *Tc*-Prd, with only 3 amino acid differences in the N-terminal portion of the PD, and is 84 % identical to that of *Dm*-Prd. The *Dmac*-Prd HD is 98 % identical to that of *Tc*-Prd, with only the most C-terminal amino acid different, and 92 % identical to the *Dm*-Prd HD. Blastx searches using sequences of other TA cloning products identified orthologs of *gsb* and *gsb*-*n* in that their predicted protein sequences possess a PD, a HD and a Gsb- or Gsb-n-type OP (*gsb* and *gsb-n* GenBank Accession number KT875128 and GenBank Accession number KT875129; Additional file 6).

### *Dmac-prd* is expressed in stripes

**Fig. 3:**
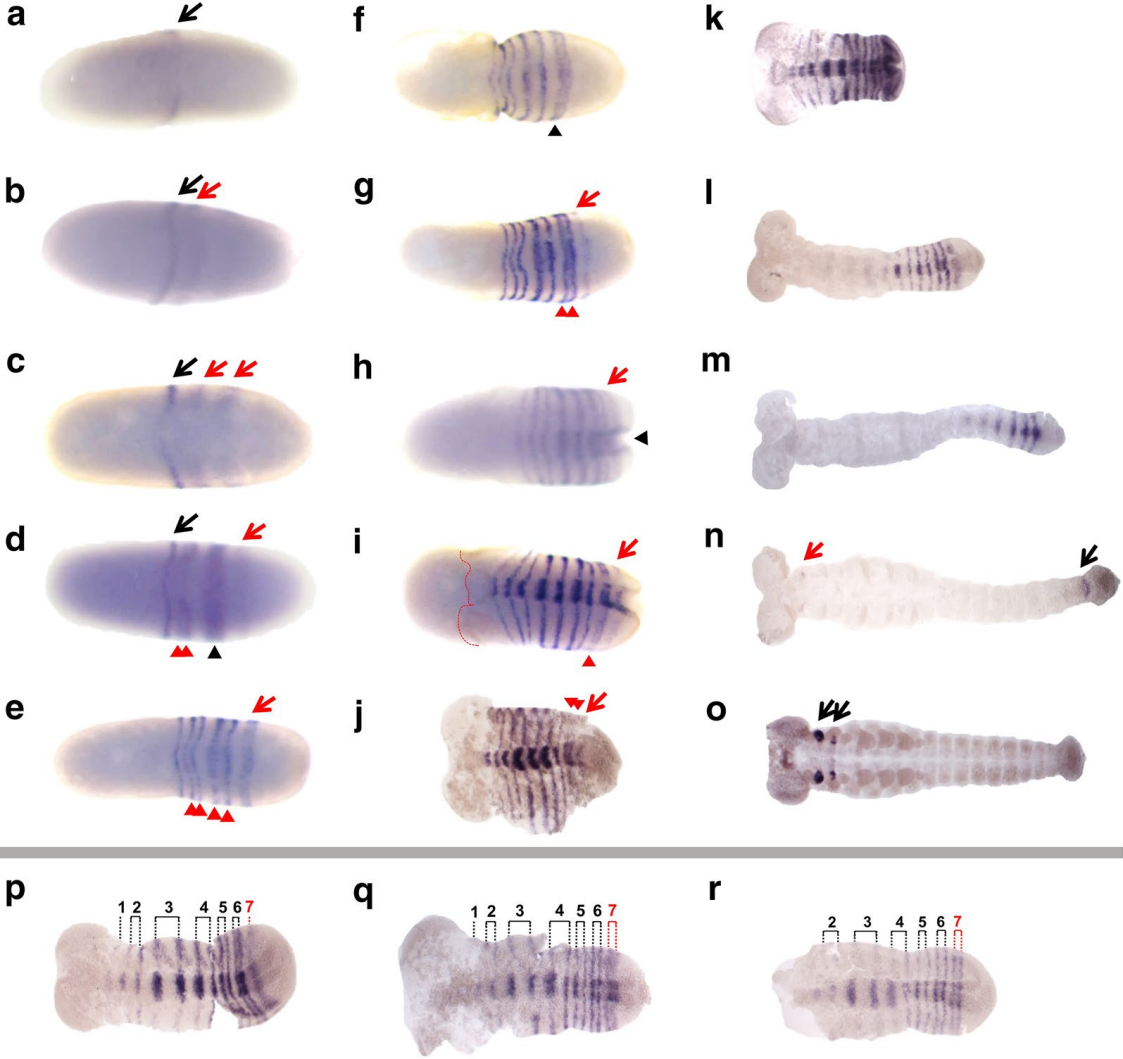
*Dmac*-*prd* is expressed in stripes during embryogenesis. Expression of *Dmac*-*prd* examined by in situ hybridization. *Arrows* and *arrowheads* indicate primary and secondary stripes, respectively. *Black arrows* show “old” primary stripes while *red arrows* indicate “new” primary stripes. *Black arrowheads* show splitting primary stripes, and *red arrowheads* indicate resolved secondary stripes. **a** A single weak stripe in early blastoderm (*black arrow*). **b** The first stripe becomes clearly detectable (*black arrow*). The second stripe emerges posterior to the first stripe (*red arrow*). **c** Two broad primary stripes appear (*red arrows*). **d** A late blastoderm stage embryo. The first primary stripe remains undivided (*black arrow*). The second primary stripe has divided into two secondary stripes (*red arrowheads*). The third primary stripe is splitting (*black arrowhead*). The fourth primary stripe is showing up *de novo* (*red arrow*). **e** When the broad shallow ventral furrow appears, the first undivided stripe, four secondary stripes (*red arrowheads*) and a fourth primary stripe are detected (*red arrow*). **f** Fading expression is detected in the center of the newly arisen stripe (*black arrowhead*). **g** The fourth primary stripe has divided into two stripes (*red arrowheads*). A weak fifth stripe appears (*red arrow*). **h** During gastrulation, a total of 8 *Dmac*-*prd* stripes are detectable. *Black arrowhead* indicates the posterior end of the ventral furrow. *Red arrow* indicates the posterior-most *Dmac*-*prd* stripe. **i**, **j** As gastrulation proceeds, a 6th primary stripe arises; bilateral head lobes become visible. *Red arrowheads* indicate the dividing stripe. *Red arrow* indicates the newly emerged stripe. *Red dashed line* in **i** shows the anterior edge of the germ rudiment. **k** Embryo during early germ band elongation with striped *Dmac*-*prd* expression across the whole germ band. **l**, **m** Elongating embryo with faint *Dmac*-*prd* stripes in anterior segments. Posterior segments have strong striped *Dmac*-*prd* expression. **n** Embryo at late germ band elongation stage. Stripes have faded except for the most posterior segment (*black arrow*). Hint of *Dmac*-*prd* expression appears in the mandibles (*red arrow*). **o** Later embryo showing *Dmac*-*prd* expression in the head (*black arrows*). **p**–**r** Detailed view of stripe splitting. **p** A total of 7 primary stripes have developed. The first stripe remains undivided. The next 5 primary stripes have resolved to secondary stripes. The 7th primary stripe emerges from the anterior region of the posterior end of the embryo as a broad weak stripe. **q** Anterior striped expression fades. The expression in the center of the 7th stripe becomes fuzzy and faint. **r** The 7th stripe has divided into two thin secondary stripes as there is no expression in the center. All embryos are shown with anterior to the *left*

To investigate the expression of *prd* in *D. maculatus*, RNA in situ hybridization in early embryos was performed. No specific staining pattern was detectable using a sense probe (data not shown). Using an antisense probe, *Dmac*-*prd* transcripts were initially detected as a single stripe at approximately 50 % of the blastoderm length (black arrow, Fig. 3a). Posterior *Dmac*-*prd* stripes emerged sequentially in an anterior to posterior fashion (Fig. 3b–n). The first primary *Dmac*-*prd* stripe resolved into a more clearly detectable thin stripe and remained undivided (black arrow, Fig. 3b, c). The second and the third primary *Dmac*-*prd* stripes first appeared as weak broad stripes in the posterior half of the embryo (red arrows, Fig. 3b, c). These two primary stripes split into pairs of thin secondary stripes (red arrowheads, Fig. 3d, e). By the time the fourth primary *Dmac*-*prd* stripe arose in the posterior region (late cellular blastoderm, red arrow, Fig. 3d), the second primary stripe had completed its split into two secondary stripes (red arrowheads, Fig. 3d), and the third primary stripe began to split (black arrowhead, Fig. 3d). At the onset of gastrulation when the ventral furrow emerged, the anterior-most undivided *Dmac*-*prd* stripe, four anterior secondary stripes and a fourth primary stripe were clearly observed (Fig. 3e).

During gastrulation, when the ventral furrow had invaginated further into the yolk and several transverse folds appeared, the fourth primary stripe had resolved into secondary stripes (arrowheads, Fig. 3f, g) and a fifth primary stripe was detected (red arrow, Fig. 3g). When the posterior invagination and the ventral furrow became more prominent (black arrowhead), a total of eight *prd* stripes (5 primary stripes, among which the first remained undivided, three middle stripes split into 6 secondary stripes, and a fifth newly arisen stripe, red arrow) were detected (Fig. 3h). Anterior stripes started to fade while posterior stripes were embedded into the posterior end due to the SAZ invagination (Fig. 3i, j). As gastrulation proceeded, the embryonic rudiment with bilateral head lobes was clearly distinguishable from the extraembryonic tissue (Fig. 3i, j, red dashed line in i indicates the anterior boundary of the germ rudiment). In the embryonic rudiment, secondary stripes resolved from the fifth primary stripe (red arrowheads, Fig. 3i, j) and a weak sixth primary stripe (red arrow, Fig. 3i, j) was detected.

As the germ band extended, new *prd* stripes arose from the region anterior of the SAZ and resolved into thin secondary stripes by fading expression in the center (Fig. 3k–n and 3p–r), as reported in other species [26, 53–55]. There was no obvious intensity or width difference within pairs of *Dmac*-*prd* secondary stripes in the blastoderm or the germ band (Fig. 3e–g and p–r). As posterior *Dmac*-*prd* stripes were added sequentially, anterior *prd* stripes became weak and eventually invisible (Fig. 3k–n). Gnathal, thoracic and abdominal *prd* stripes disappeared gradually during germ band extension (Fig. 3l–n). During later embryogenesis, *prd* was strongly expressed in appendage primordia in gnathal segments (black arrows, Fig. 3o). Together, the conserved protein sequence, expression in stripes, and the characteristic splitting of primary stripes into secondary stripes in early embryos, suggested that *Dmac*-*prd* is involved in pair-rule patterning. The finding that a total of four primary *Dmac*-*prd* stripes are present at the onset of gastrulation is consistent with the assignment of *D. maculatus* as an intermediate-germ insect.

### RNAi knockdown of *Dmac-prd* results in defects in segmentation

**Fig. 4:**
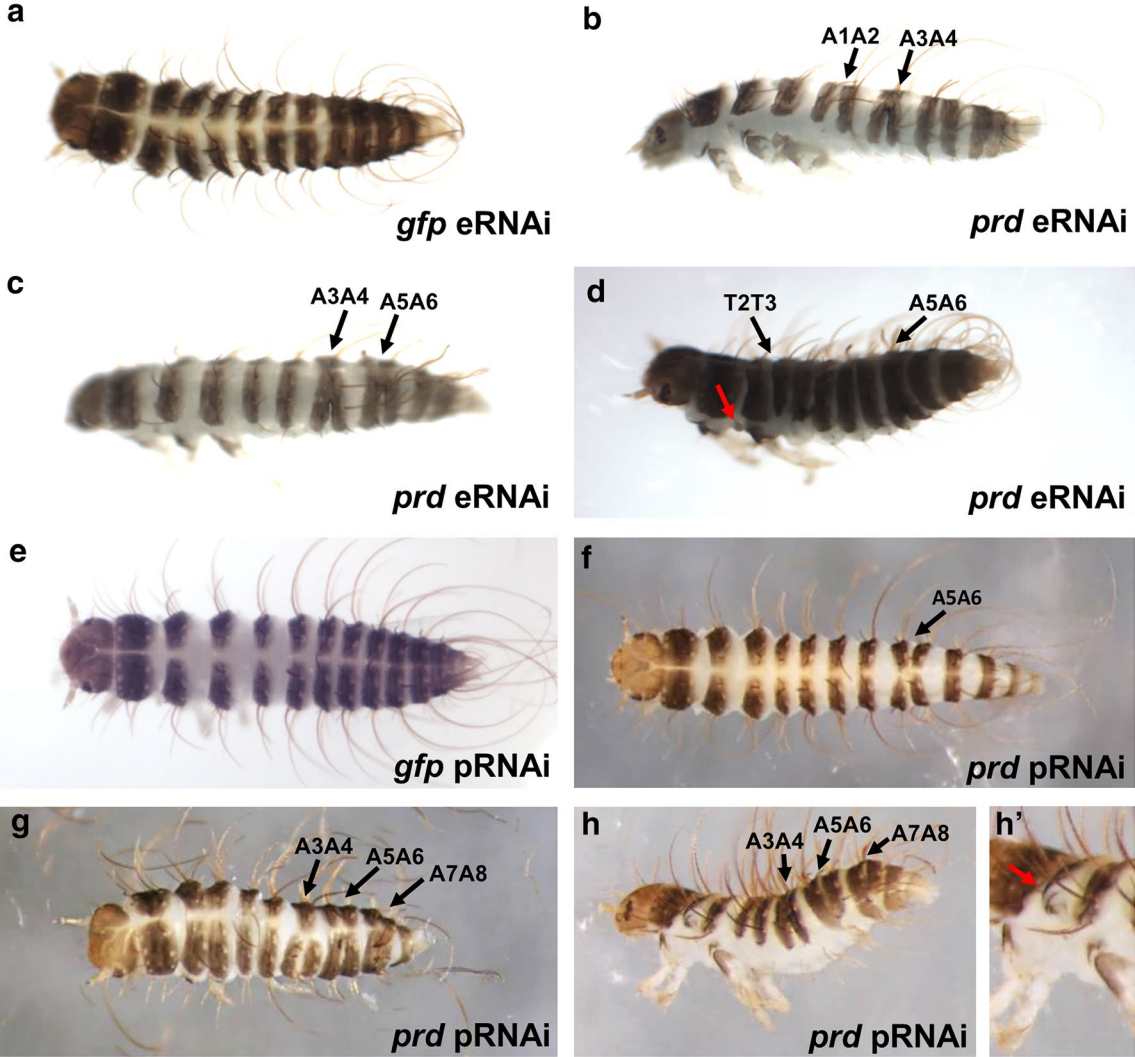
Knockdown of *Dmac*-*prd* with RNAi causes segmentation defects. *Dmac*-*prd* or *gfp* RNAi was carried out, as indicated. *gfp* dsRNA was injected as negative control. **a**–**d** Embryonic RNAi. **e**–**h** Parental RNAi. **a** Dorsal view of a first instar *D. maculatus* larva after *gfp* dsRNA injection showing wild-type phenotype with head, three thoracic segments and ten abdominal segments. **b** A hatched first instar larva after *Dmac*-*prd* dsRNA injection contains fused A1/A2 and A3/A4 segments (*black arrows*). **c** Lateral view of a larva with fused A3/A4 and A5/A6 segments after *Dmac*-*prd* eRNAi (*black arrows*). **d** T2 legs are missing in hatched larva with severe phenotype after *Dmac*-*prd* eRNAi (*red arrow*). *Black arrows* indicate fused T2/T3 and A5/A6 segments. **e** Offspring produced by *gfp* dsRNA injected female are viable until hatching and show wild-type phenotype (*dorsal view*). **f** Dorsal view of a hatched offspring with fused A5/A6 segments from *Dmac*-*prd* 3′ dsRNA injected female (*black arrow*). **g**, **h** First instar larva after *Dmac*-*prd* (3′ and 5′, respectively) pRNAi with shortened body length as well as fused segments. *Black arrows* indicate fusions of adjacent segments. **h**’ *Red arrow* indicates defective T2 leg

To investigate the function of *prd* in *D. maculatus*, and to determine whether RNA interference (RNAi) is effective in this species, we performed embryonic RNAi (eRNAi). *Dmac*-*prd* 3′ dsRNA, corresponding to a 254 bp region downstream of the HD, was injected into pre-blastoderm stage embryos (target region is indicated in Fig. 2). After injection, all hatched offspring from control embryos injected with *gfp* dsRNA were wild type in appearance with head, three thoracic segments and ten abdominal segments (Fig. 4a). In contrast, over 85 % (18/21) of the newly hatched larvae after *Dmac*-*prd* 3′ dsRNA injections showed segmentation defects with one or several fused segmental boundaries (T2/T3, A1/A2, A3/A4, A5/A6, A7/A8; black arrows in Fig. 4b–d), reminiscent of the segmentation phenotype produced by *eve* eRNAi in cricket [56]. Some cases included loss of or abnormal development of T2 legs (red arrow, Fig. 4d).

**Fig. 5:**
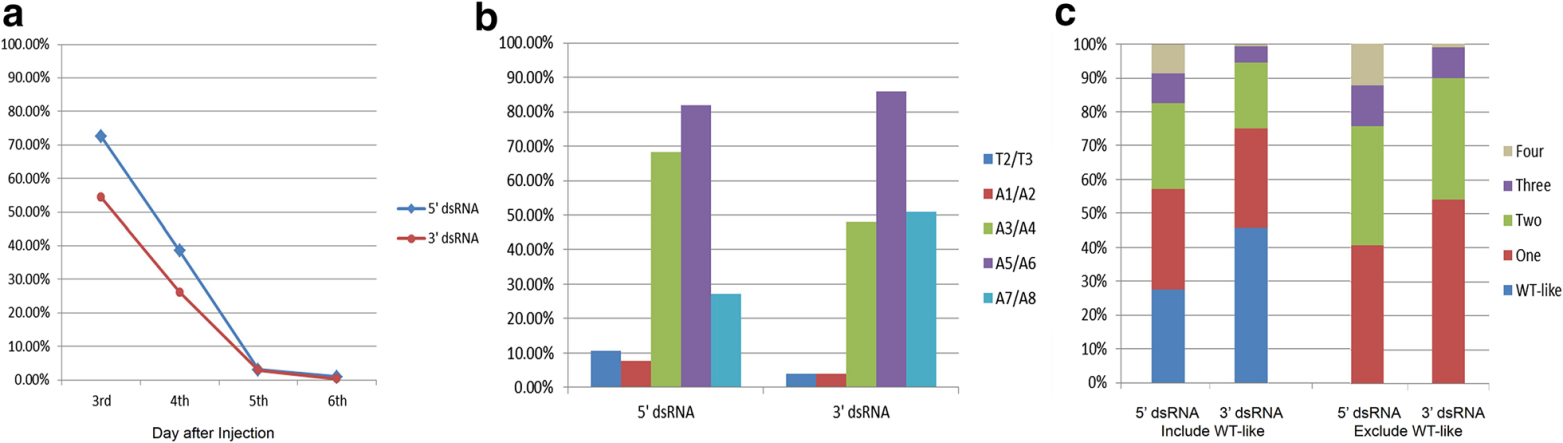
Quantitation of *Dmac*-*prd* pRNAi segmentation defects. Offspring produced by twelve of either *Dmac*-*prd* 5′ or 3′ dsRNA injected females, as indicated. Each hatched larva was stretched out using tweezers and examined under a dissecting microscope. **a** Percent of hatched pRNAi offspring showing segmentation phenotypes. **b**–**c** Embryos were collected on the third day after injection and segmentation defects were scored. **b** Frequency of fusion of specific pairs of adjacent segments. Note that some larvae had more than one pair fused. **c** Frequency of types of segmentation defects observed. *Left bars* percentage of hatched larvae with wild-type segmentation, and those displaying one, two, three or four segmental fusions; *right bars* percentage of hatched larvae with one, two, three or four segmental fusions among those with observable segmentation defects

Injection of dsRNA into pupal or adult females can result in phenotypes evident in their offspring. This phenomenon was named parental RNAi (pRNAi) and has been observed in *T. castanum*, *O. fasciatus*, *Gryllus bimaculatus*, *Blattella germanica,N. vitripennis,* and other species [57–61]. To determine whether pRNAi functions in *D. maculatus,* and to verify the segmentation phenotypes observed with *Dmac*-*prd* eRNAi, *Dmac*-*prd* 3′ dsRNA was injected into newly eclosed virgin females and their offspring were examined. To ensure specificity, a second dsRNA was generated from a non-overlapping target region (*Dmac*-*prd* 5′, 256 bp; Fig. 2). There was no significant difference in the offspring yield or hatch rates between *gfp* dsRNA injected and *Dmac*-*prd* 5′ or 3′ dsRNA injected females (data not shown). Segmentation in all hatched offspring from control females injected with *gfp* dsRNA appeared to be wild type (Fig. 4e). In contrast, over 50 % (100/184) of hatched offspring collected on the 3rd day after injection from *Dmac*-*prd* 3′ dsRNA injected females and ~73 % (66/91) from *Dmac*-*prd* 5′ dsRNA injected females displayed segmentation defects (Figs. 4f–h, 5a). The percentage dropped to less than 30 % (51/195) and ~39 % (52/135) on the 4th day after injection for *Dmac*-*prd* 3′ and *Dmac*-*prd* 5′, respectively (Fig. 5a). On the 5th day after injection, less than 3 % of embryos hatched with segmentation defects (*Dmac*-*prd* 5′, 6/202; *Dmac*-*prd* 3′ 9/305; Fig. 5a). Only very few embryos collected on the 6th day after injection hatched with fused segments (2/234, *Dmac*-*prd* 5′; 1/282, *Dmac*-*prd* 3′; Fig. 5a).

Analysis of segmentation defects revealed a range of defects, phenocopying an allelic series. In mildly affected larvae, partial or complete fusion was observed for one pair of adjacent segments, most often A5/A6 (Figs. 4f, 5b). In other cases, fusions were detected between two, three or four adjacent segments (Figs. 4g, h, 5c). The fusions occurred in the same alternating fashion as observed for eRNAi (Figs. 4f–h, 5b). Missing or defective T2 legs were also observed in some severe cases. Very often, the defective T2 legs projected from the lateral edge of instead of the ventral-lateral side of the T2 segment (red arrow, Fig. 4h’).

To further analyze the role of *Dmac*-*prd* in segmentation, defects were quantitated in hatched embryos collected on the third day after injection. More than 70 % of *Dmac*-*prd* 5′ dsRNA offspring displayed some type of defect (Fig. 5a). Of these, ~40 % displayed one segmental fusion while nearly 60 % hatched with more than one segment fused (35 % with two fusions, 12 % with three fusions and 12 % with four fusions; Fig. 5c). The percentage of *Dmac*-*prd* 3′ dsRNA affected offspring was over 50 % (Fig. 5a). Of these, 54 % had one segmental fusion, 36 % had two, 9 % had three, and 1 % had four segments fused (Fig. 5c). Overall, segments A5/A6 were most commonly affected by *Dmac*-*prd* knockdown, with over 80 % of either *Dmac*-*prd* 5′ or 3′ dsRNA affected larvae displaying fusion of these segments (Fig. 5b). Fusion of A3/A4 was seen in 68 % and 48 % of *Dmac*-*prd* 5′ and 3′ affected larvae, respectively. Fusion of A7/A8 was detected in 27 and 51 % of *Dmac*-*prd* 5′ and 3′ affected larvae, respectively. Fusions of T2/T3 and A1/A2 had lower frequencies (11 and 4 % for fused T2/T3 in *Dmac*-*prd* 5′ and 3′ affected larvae, respectively; 8 and 4 % for fused A1/A2 in *Dmac*-*prd* 5′ and 3′ affected larvae, respectively). These differences in frequency suggest differential susceptibility of different parasegments to *Dmac*-*prd* knockdown (Fig. 5b).

In sum, both eRNAi and pRNAi were effective tools to analyze gene function in *D. maculatus*. Analysis of the morphology of larvae hatched after knockdown of *Dmac*-*prd* indicates a role for *prd* in segmentation in this species.

### *Dmac-prd* is necessary for the expression of alternate Engrailed stripes

**Fig. 6:**
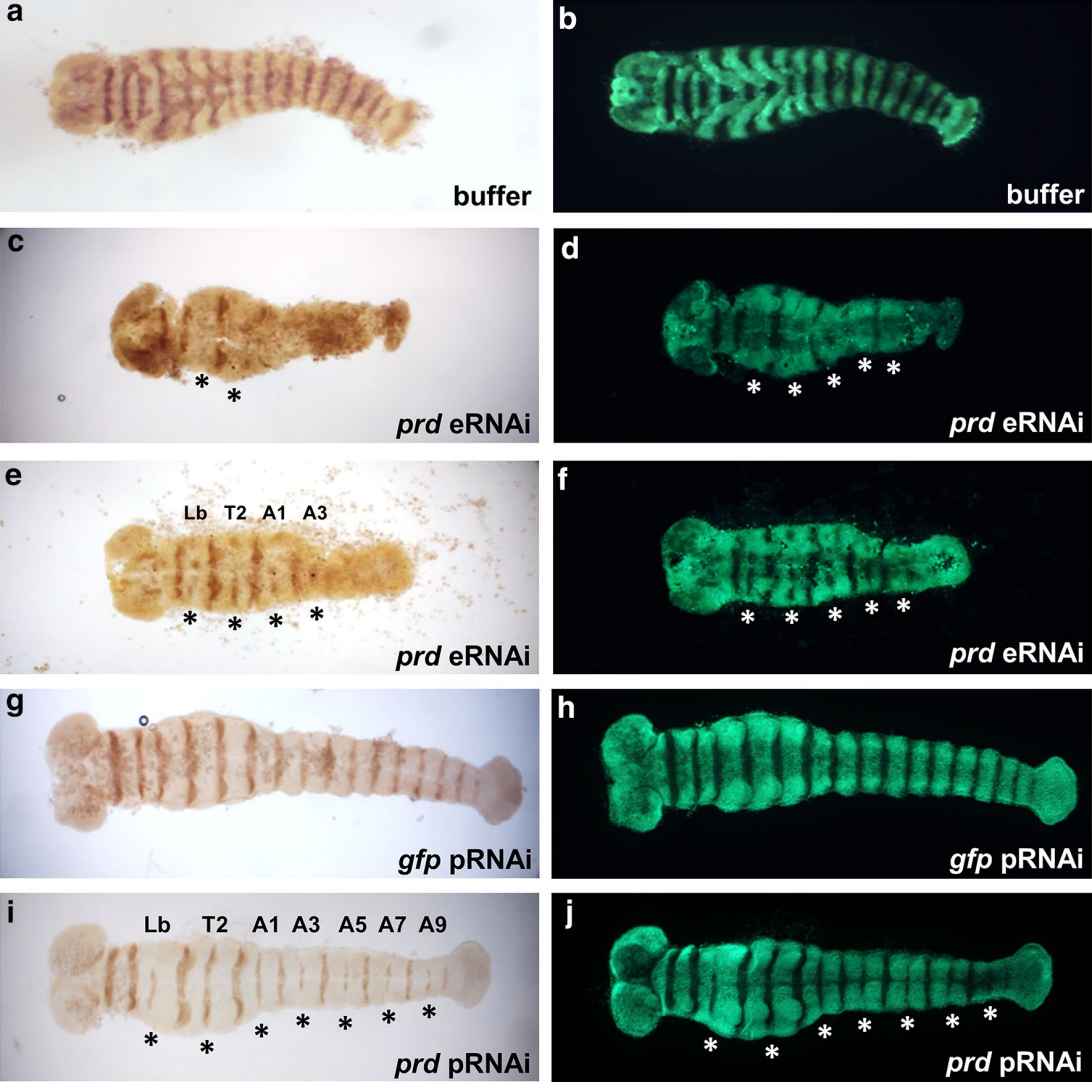
Reduced expression of alternate Engrailed stripes after *Dmac*-*prd* RNAi. **a**–**f** Embryonic RNAi. **g**–**j** parental RNAi. **a**, **c**, **e**, **g**, **i** Injected embryos 24–27 h AEL (eRNAi) or 0–1 d AEL embryos from injected females (pRNAi), as indicated were fixed and stained using anti-En 4D9 primary antibody and DAB staining. **b**, **d**, **f**, **h**, **j** SYTOX Green nuclear staining of same embryos for visualization of morphological defects. *Asterisks* indicate reduced En expression, fused segments or partial fusion between two neighboring segments

In both *D. melanogaster* and *T. castaneum*, *prd* functions as a pair-rule gene and regulates *en* expression in odd-numbered segments [26, 62]. We therefore asked if *Dmac*-*prd* functions similarly to regulate the expression of alternate En stripes in *D. maculatus*. Embryos injected with buffer alone showed equally strong En expression in every segment (Fig. 6a). In contrast, loss of En expression in alternating segments was evident in over 50 % (25/46) of extended germ bands after *Dmac*-*prd* eRNAi (asterisks, Fig. 6c, e). Germ band morphology was also analyzed using nuclear staining with SYTOX Green. This revealed partial or even complete fusion of pairs of adjacent segments into a wider segment (asterisks, Fig. 6d, f).

Since injection of embryos may have caused damage that precluded a more careful analysis of En expression, embryos laid by *Dmac*-*prd* dsRNA-injected females were also examined. While offspring from the *gfp* dsRNA control injected females displayed wild-type-like En expression (Fig. 6g), loss of or reduced En expression in the labium, T2, A1, A3, A5, A7 and A9 segments were detected in over 60 % (112/179) of extended germ band stage embryos from *Dmac*-*prd* dsRNA injected females (asterisks, Fig. 6i). Segmental fusion was observed in the posterior region of odd-numbered segments following nuclear staining in the regions where loss of En expression was detected (asterisks, Fig. 6j).

The decreased expression of alternate En stripes, as well as the segmentation defects observed in embryos in which *Dmac*-*prd* was knocked down, indicate that *Dmac*-*prd* functions as a pair-rule segmentation gene in *D. maculatus*.

## Discussion

Here we have established *D. maculatus* as a new system for studying embryonic development, gene expression and gene function. *D. maculatus* were maintained in long-term culture in the lab and large numbers of embryos were readily collected and processed. The timing and progression of nuclear divisions, cellularization, gastrulation, and germ band development were described (Fig. 1; Additional file 4). Genes of the *Pax3/7* family were isolated (Fig. 2; Additional file 6) and the *Dmac*-*prd* ortholog was found to be expressed in stripes in blastoderm, gastrulation and germ band extension stages embryos, with additional stripes added from the posterior region (Fig. 3). Both eRNAi and pRNAi were effective in this species, revealing a role for *Dmac*-*prd* in pair-rule patterning (Figs. 4, 5, 6), similar to that seen in other insects [25, 26, 53, 54, 63, 64]. These findings suggest that the role of *prd* in pair-rule patterning is shared among holometabolous insects with different modes of embryonic development.

Our studies support the classification of *D. maculatus* as an intermediate-germ beetle, as four primary *prd* stripes were established in late blastoderm (Fig. 3d). In contrast, only one Prd/*prd* stripe was seen in *T. castaneum* embryos prior to gastrulation [26, 38]. In *T. castaneum*, the pair-rule segmentation genes *hairy* and *even*-*skipped* (*eve*) are expressed in two stripes before gastrulation [17, 23, 26, 32, 65]. One En and one *wingless* stripe were detected at the same stage [17, 32, 66, 67]. In a long-germ beetle, *Callosobruchus maculatus*, six *eve* primary stripes were evident before gastrulation [32], while four *eve* primary stripes were present in late blastoderm *Dermestes frischi* embryos [32], similar to what we observed for *prd*.

To our knowledge, this is the first demonstration of RNAi function in dermestids. Dermestid beetles include 500–700 species worldwide. *D. maculatus* (common name hide or skin beetle), has been widely used for skeletonizing dead animals [68]. It is a worldwide pest for the stored meat industry and also the silk industry because it slaughters silkworm cocoons [69, 70]. Various dermestid species feed on stored meat, stored grain, silk, cheese, poultry, natural or synthetic fiber and pollen [69]. Because of their large numbers and their ability to occupy such diverse habitats, different beetle species have become economically significant pests for agriculture, forests, fabric, and stored food supplies, thus impacting both households and industry [7, 71]. The use of RNAi as a highly specific and safe method to control insect pests shows promise in a number of different taxa [72]. Our studies suggest that RNAi will be a viable strategy for control of dermestid pests.

### Protein motifs mix and match in Pax family members

*prd* was the founding member of the metazoan Pax family of transcription factors, part of the genetic toolkit directing animal development [39, 48, 73]. Pax family members have taken on diverse roles in embryonic development, organogenesis and have been implicated in a number of human cancers [51, 74–76]. Pax family proteins are characterized by the presence of multiple protein domains, including a paired domain (PD) composed of a bipartite DNA-binding domain (PAI and RED domains separated by a linker region), an octapeptide (OP), and a paired-type homeodomain (PTHD) [77]. Members of different Pax subfamilies contain different combinations of these protein domains, or even truncated versions of individual domains, imparting diversity in both structure and function to this gene family [39, 40, 48, 78–82]. For example, *D. melanogaster* Pox-meso and Pox-neuro have the PD but lack a HD [78]. Phylogentic analyses suggest that *Pax* genes fall into distinct subfamilies, with *prd* a member of the *Pax3/7* group [81, 82]. Pax3/7 family members generally contain a PD, OP and HD and are represented by both *prd* and the closely related *gsb* and *gsb*-*n* genes in *D. melanogaster*, with only *prd* involved in pair-rule segmentation in *D. melanogaster* [39–41, 48, 49, 53, 63, 83]. Although both the PD and HD are shared by all these genes, the OP is present in Gsb and Gsb-n but not in Prd [40, 48]. Similarly, in *T. castaneum* and *A. mellifera*, the OP motif is present in Gsb and Gsb-n but not in Prd and also is not found in the only *N. vitripennis* Pax3/7 family member [26, 50]. However, the OP is found in many other Pax proteins: e.g., insect Gsb/Gsb-n, Shaven, Pox-meso and Pox-neuro [50] and mammalian Pax 1/9 and Pax 2/5/8 [84]. Phylogenetic analysis suggests that the OP was a feature of ancestral Pax proteins. The presence of the OP in Gsb and Gsb-n but not in Prd of extant insects suggests that during Pax3/7 evolution, the OP was lost in an ancestral Prd ortholog. Therefore, the absence of the OP serves as a signature motif for identification of *prd* orthologs [50, 51]. In this study, of the three *prd* family member genes isolated, only one lacked the OP (Fig. 2; Additional file 6). Expression and functional results demonstrated it to be a bona fide *prd* ortholog (Figs. 3, 4, 5, 6), consistent with the utility of using the OP motif as a signature to distinguish among *prd* family members.

### *Pax3/7* function in panarthropods

*Pax3/7* family members have been isolated from a broad range of arthropod groups and from the outgroups, Onychophora and Tardigrada. Expression studies suggest a conserved role in segmentation with segmentally expressed stripes seen for *Pax3/7* genes from crustaceans, chelicates, myriapods, two onychophorans and a tardigrade, suggesting that the ancestral function in segmentation was of the segment polarity type, affecting every segment [13–16, 85–87]. Indications of a pair-rule type expression are seen in the millipede, *Glomeris marginata*, where the *Pax3/7* family gene *pairberry1* (*pby*-*1)* is expressed in stripes in the head and anterior thorax. Although these stripes arise almost simultaneously, their intensity alternates in every other segment [13, 88]. In the two-spotted spider mite (Chelicerata: *Tetranychus urticae*), the delayed appearance of alternating stripes of a *Pax3/7* is reminiscent of pair-rule-type expression [86, 89]. However, it is only in Pancrustacea, or possibly hexapods, that a clear PR-like expression pattern of *Pax3/7* genes is observed [38]. The *Schistocerca americana* ortholog *pby*-*1* is expressed in a pair-rule-like pattern before it is expressed segmentally [38]. Although a role for Pax3/7 in PR patterning may thus have arisen before the origin of holometabolous insects, it is in this clade that PR expression and function has been most extensively documented.

A detailed comparison of the expression of *Dmac*-*prd* to that seen for *prd* in other holometabolous insects shows similarities and differences within this large clade. *Dmac*-*prd* expression is initiated as a single stripe in the blastoderm (Fig. 3a). In *T. castaneum*, *prd* expression also begins as a single stripe in the presumptive mandibular segment [26]. Unlike *prd* in these two beetles, *D. melanogaster* Prd first is expressed in a broad anterior region that then resolves into a broad stripe [53]. Posterior *Dmac*-*prd* stripes appear sequentially in an anterior to posterior fashion in the blastoderm embryo to generate a total of 4 primary stripes before gastrulation (Fig. 3d). Sequential addition of *prd* stripes in the blastoderm was also detected in *A. mellifera* and *N. vitripennis* [50, 55]. This anterior to posterior progression of stripe formation in the blastoderm was also reported for other pair-rule genes in *T. castaneum*, *N. vitripennis a* and *O. fasciatus* [22, 23, 29]. In contrast to this, in *D. melanogaster*, the primary Prd stripes 4 and 7 are expressed earlier than stripes 3, 5, 6 and 8 [53]. Thus, even though Prd stripes do not appear simultaneously in long-germ *D. melanogaster*, they do not arise sequentially from the posterior end.

The remaining primary *Dmac*-*prd* stripes are added from the posterior region during germ band elongation (Fig. 3), as in *T. castaneum* [26, 38]. As in other species, including *D. melanogaster*, the primary *prd* stripes in *D. maculatus* split into two secondary stripes (Fig. 3e–g, p–r). As seen in *A. mellifera*, we did not detect any difference in the intensity or width within pairs of stripes, although differences were reported for *T. castaneum*, *N. vitripennis* and *D. melanogaster* [26, 38, 50, 53–55]. Therefore, to date, there is no obvious correlation between this feature and germ band mode. During germ band elongation, anterior *Dmac*-*prd* stripes fade while stripes in posterior abdominal segments display strong expression (Fig. 3k–n). This feature is shared in *T. castaneum* and *A. mellifera* [26, 55], but equally expressed segmental *prd* stripes without fading of anterior stripes were observed in late blastoderm and fully elongated *N. vitripennis* and *D. melanogaster* germ band embryos [50, 53]. Since *A. mellifera*, *N. vitripennis* and *D. melanogaster* exhibit a long-germ mode of segmentation, while *T. castaneum* and *D. maculatus* show short- and intermediate-germ modes, such fading of anterior *prd* stripes during later embryogenesis cannot be correlated with germ band mode. Later during development, *Dmac*-*prd* is strongly expressed in gnathal segments (Fig. 3o). This late *prd* expression pattern appears to be a common feature in insects examined so far, suggesting a conserved function for *prd* in head development [50, 53–55, 90, 91]. In sum, although there is some divergence suggesting subtle modulation of *prd* expression, the early striped expression, the splitting of primary *prd* stripes, and the late head expression appear to be shared throughout insect taxa.

### *Dmac-prd* functions as a pair-rule gene

As seen in other RNAi knockdown experiments, both *Dmac*-*prd* pRNAi and eRNAi resulted in a graded series of defects. Two non-overlapping target regions were used to perform pRNAi and both gave similar results, suggesting that effects were specific. In pRNAi experiments, the penetrance dropped rapidly within one-week of injection (Fig. 5a). pRNAi in *T. castaneum* displayed relatively high penetrance after weeks [58]. Whether this difference is specific to *Dmac*-*prd* or a general feature of RNAi in *D. maculatus* remains to be determined.

Both eRNAi and pRNAi produced defective larvae with fused segmental boundary/boundaries between T2/T3 (parasegment 5, ps5), A1/A2 (ps7), A3/A4 (ps9), A5/A6 (ps11), A7/A8 (ps13). In this graded series, larvae displayed segmentation defects with different levels of severity (Figs. 4, 5c). One parasegment (A5/A6) was more sensitive to RNAi, even with low levels of knockdown (Fig. 5b), as has also been reported in other species for pair-rule mutation or knockdown [29, 92]. En expression was reduced or completely lost in odd-numbered segments in ~50 % of *Dmac*-*prd* dsRNA injected embryos and ~60 % of pRNAi offspring (Fig. 6). Together, these findings suggest that *Dmac*-*prd* functions as a pair-rule segmentation gene in odd-numbered parasegments by activating *en* expression. This function is shared with shorter-germ *T. castaneum* and long-germ *D. melanogaster* [25, 26, 62, 64], and thus appears to be conserved, irrespective of the mode of segmentation.

## Conclusions

Here we have established basic approaches necessary to use *D. maculatus* as a new insect model system. Methods are available not only for basic research approaches but also for developing alternative and safe methods for control of dermestid pests. *D. maculatus* represents the diverse clade of Coleoptera and displays an intermediate-germ mode of segment addition, making it a good system for comparative studies with shorter-germ *T. castaneum* and long-germ *D. malanogaster*. These comparative studies were initiated here by the isolation and characterization of the *D. maculatus* ortholog of *prd*. Consistent with the role of *prd* in *D. melanogaster* and *T. castaneum, prd* functions as a pair-rule segmentation gene in *D. maculatus.* Thus, *prd* appears to be a ‘core’ pair-rule gene that retains pair-rule function in a range of insects that display variation in the function of other pair-rule genes and in the mode of segment addition.

## Additional files

10.1186/s13227-015-0028-0 COI identification of laboratory reared species. The COI gene from our lab *D. maculatus* colony was compared to the published *D. maculatus* COI sequence (GenBank ID HM909035.1). Red arrow shows mismatch. Alignment was performed using ClustalW2.
10.1186/s13227-015-0028-0 Protocols for *D. maculatus* embryo fixation and whole mount in situ hybridization.
10.1186/s13227-015-0028-0 Female and male *D. maculatus* pupae. Morphology used to distinguish female and male *D. maculatus* is shown in this photograph. (A) Two genital papillae at the posterior end of a female pupa (white arrows). (B) Male pupa has a median sternal lobe on the ventral side of the posterior abdomen (black arrow).
10.1186/s13227-015-0028-0 Gastrulation in *D. maculatus* embryos. Embryos were stained with SYTOX Green. (A) Embryo from overnight collection. Note that nuclei are closely packed together posteriorly with large and loosely arranged nuclei in the anterior dorsal region. (B-E) embryos were collected between 10 and 12 h AEL at 25 °C. Left column, lateral view; right column, ventral view of same embryo. (B, B’) The ventral furrow (vf) and several transverse folds appear as signs of early gastrulation. White arrowhead indicates the boundary between the embryo proper and extraembryonic tissue on the dorsal side. (C, C’) Ventral furrow invaginates towards the yolk. The anterior fold separates the head lobes from the anterior extraembryonic tissue. The boundary between the embryo proper and extraembryonic tissue is indicated by the white arrowhead. (D, D’) The narrower and deeper ventral furrow reaches the posterior end. The amnion folds over the posterior end of the germ rudiment, forming the posterior amniotic fold (paf). Involuting head lobes (hl) are visible. Red arrowhead shows the edge of the paf. (E, E’) The amnion, together with the serosa, moves anteriorly on the ventral side of the embryo, leaving an open serosal window (sw). Red arrowhead indicates the posterior edge of sw.
10.1186/s13227-015-0028-0 *D. mauclatus* early embryogenesis at 25 and 30 °C. Embryos were collected every 2 h AEL at 25 or 30 °C over an 18-h or a 10-h period, respectively. *D. maculatus* embryogenesis was examined using nuclear and phalloidin staining. Embryos at the end of 8–10 h AEL at 30 °C are roughly equivalent to 14–16 h AEL embryos at 25 °C.
10.1186/s13227-015-0028-0 Alignment of partial *Dmac*-Gsb, Gsb-n, and Prd protein sequences. Black lines indicate the paired domain (PD), octapeptide (OP) and homeodomain (HD). Note that *Dmac*-Prd is lacking the OP motif. Gsb has a Gsb-type OP: HSIDGILG. Gsb-n has a Gsb-n type OP: YTIDGILG. Protein sequence alignment was performed using ClustalW2. * indicates identical residue, : indicates conserved substitutions, . indicates weakly similar substitutions. Colors indicate residues are classified into groups according to their physicochemical properties. Red: Nonpolar side chain; Green: Polar side chain; Blue: Negatively charged side chain; Magenta: Positively charged side chain.

## Abbreviations

*D. melanogaster or Dm*: *Drosophila melanogaster*
*T. castaneum or Tc*: *Tribolium castaneum*
*D. maculatus or Dmac*: *Dermestes maculatus*
PRG: pair-rule gene
*prd*: *paired*
*eve*: *even*-*skipped*
COI: cytochrome c oxidase subunit I
bp: base pair
h: hour(s)
AEL: after egg laying
*gsb*: *gooseberry*
*gsb*-*n*: *gooseberry*-*neuro*
PD: paired domain
HD: homeodomain
OP: octapeptide
SAZ: segment addition zone
vf: ventral furrow
hl: head lobe
paf: posterior amniotic fold
sw: serosal window
eRNAi: embryonic RNA interference
pRNAi: parental RNA interference
dsRNA: double-stranded RNA
*en/*En: *engrailed*/Engrailed
ps: parasegment
