# Supplementary figures and images for: Dermestes maculatus: an intermediate-germ beetle model system for evo-devo
Source: EvoDevo. 2015 Oct 16;6:32. doi: 10.1186/s13227-015-0028-0 (PMC4609124; doi:10.1186/s13227-015-0028-0)

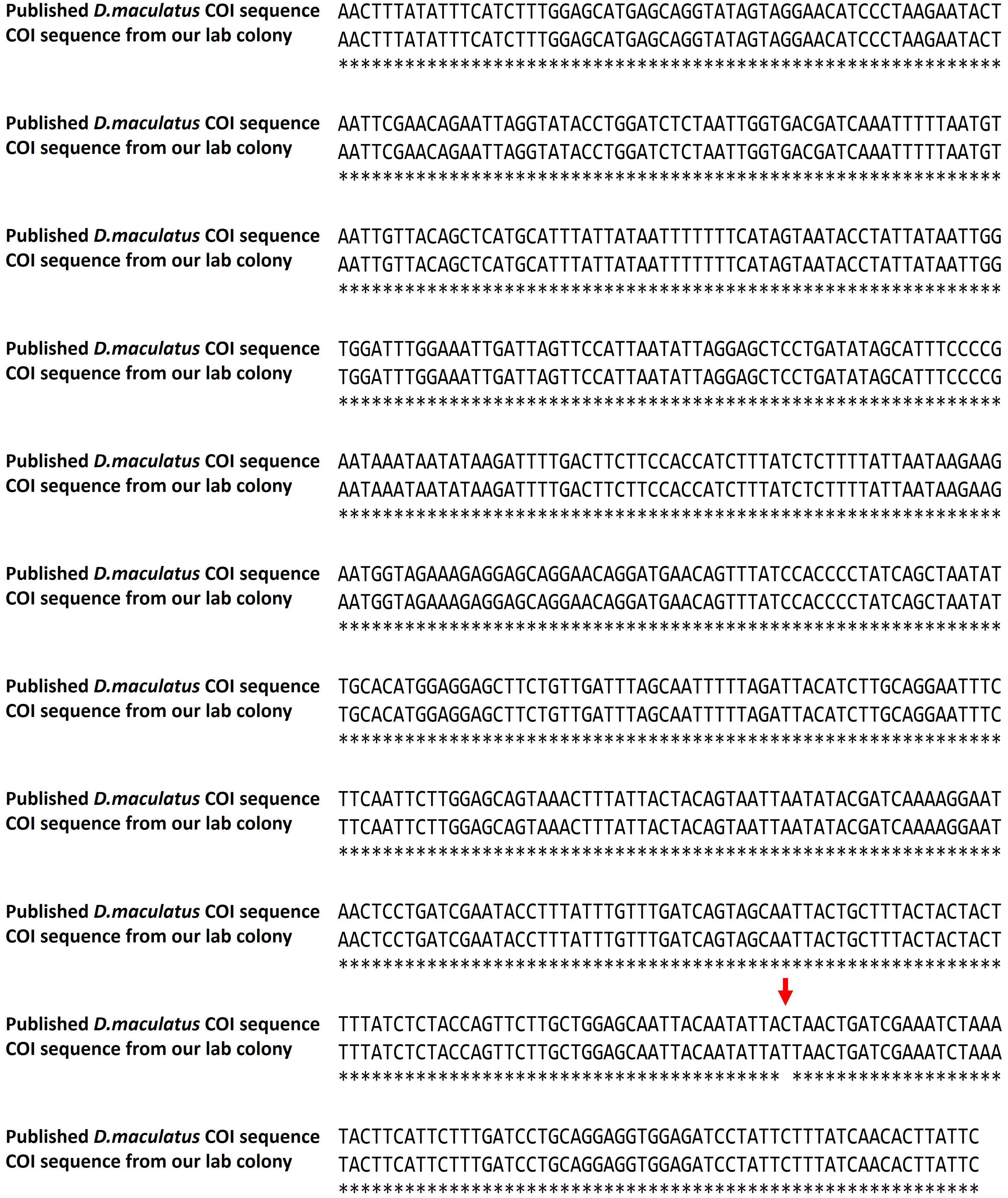

Supplement: Supplementary file 1 — 10.1186/s13227-015-0028-0 COI identification of laboratory reared species. The COI gene from our lab D. maculatus colony was compared to the published D. maculatus COI sequence (GenBank ID HM909035.1). Red arrow shows mismatch. Alignment was performed using ClustalW2. [file 13227_2015_28_MOESM1_ESM.tif]

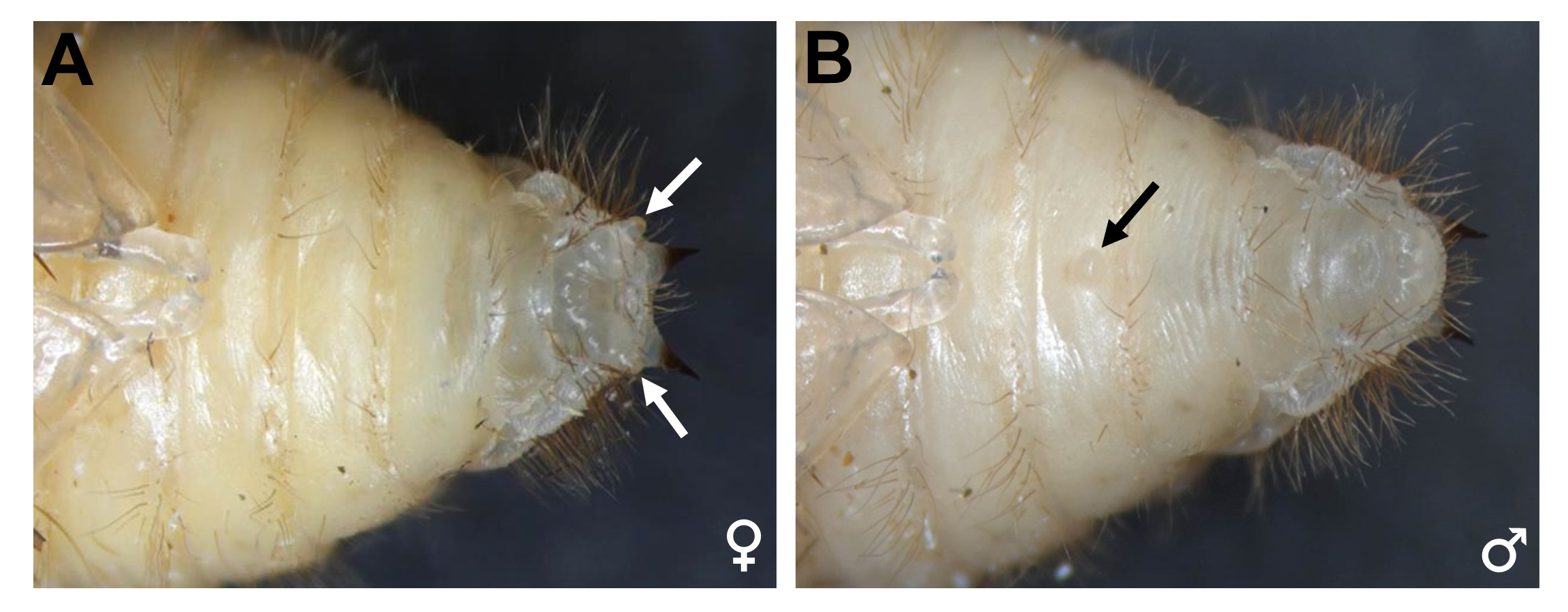

Supplement: Supplementary file 3 — 10.1186/s13227-015-0028-0 Female and male D. maculatus pupae. Morphology used to distinguish female and male D. maculatus is shown in this photograph. (A) Two genital papillae at the posterior end of a female pupa (white arrows). (B) Male pupa has a median sternal lobe on the ventral side of the posterior abdomen (black arrow). [file 13227_2015_28_MOESM3_ESM.tif]

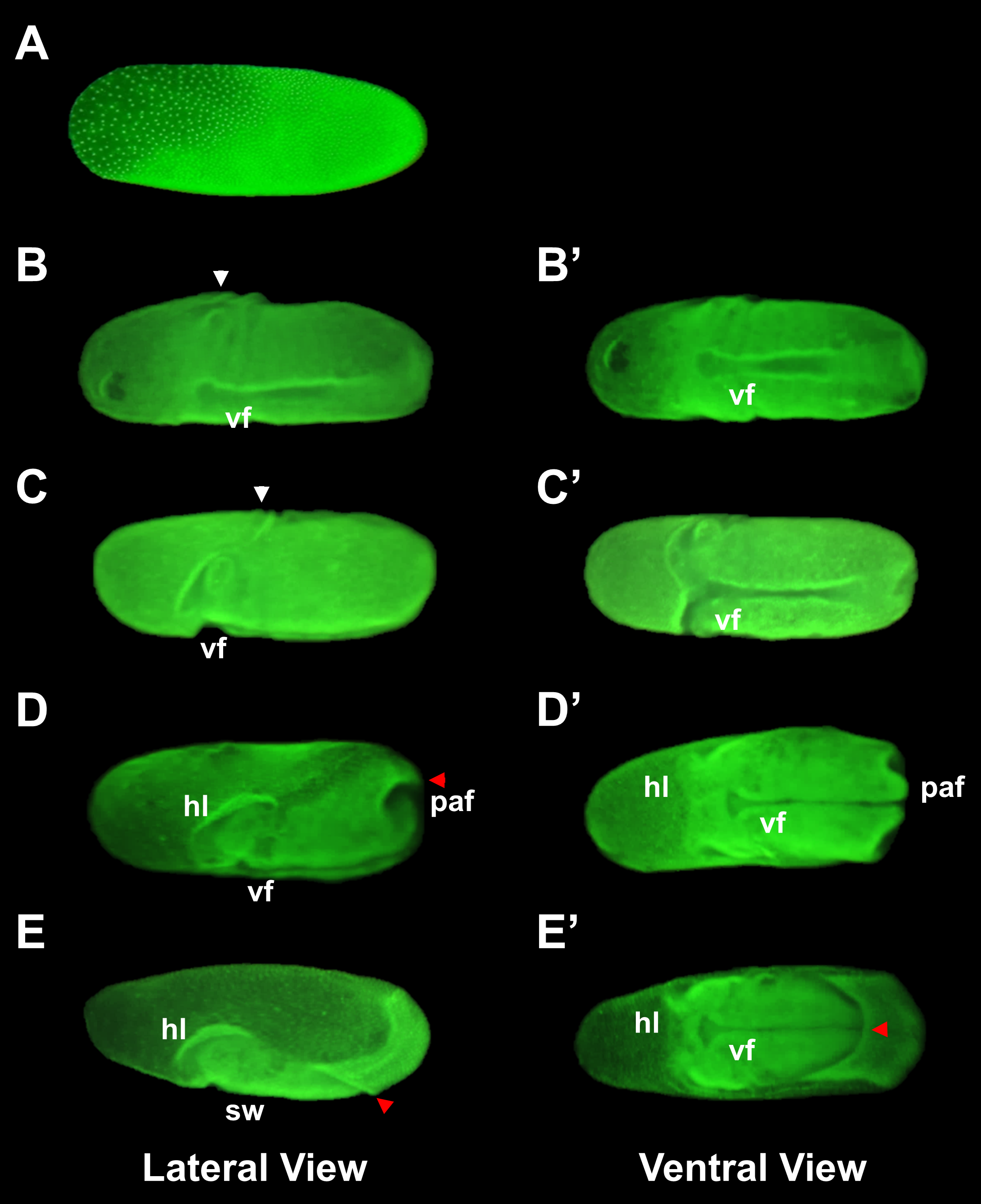

Supplement: Supplementary file 4 — 10.1186/s13227-015-0028-0 Gastrulation in D. maculatus embryos. Embryos were stained with SYTOX Green. (A) Embryo from overnight collection. Note that nuclei are closely packed together posteriorly with large and loosely arranged nuclei in the anterior dorsal region. (B-E) embryos were collected between 10 and 12 h AEL at 25 °C. Left column, lateral view; right column, ventral view of same embryo. (B, B’) The ventral furrow (vf) and several transverse folds appear as signs of early gastrulation. White arrowhead indicates the boundary between the embryo proper and extraembryonic tissue on the dorsal side. (C, C’) Ventral furrow invaginates towards the yolk. The anterior fold separates the head lobes from the anterior extraembryonic tissue. The boundary between the embryo proper and extraembryonic tissue is indicated by the white arrowhead. (D, D’) The narrower and deeper ventral furrow reaches the posterior end. The amnion folds over the posterior end of the germ rudiment, forming the posterior amniotic fold (paf). Involuting head lobes (hl) are visible. Red arrowhead shows the edge of the paf. (E, E’) The amnion, together with the serosa, moves anteriorly on the ventral side of the embryo, leaving an open serosal window (sw). Red arrowhead indicates the posterior edge of sw. [file 13227_2015_28_MOESM4_ESM.tif]

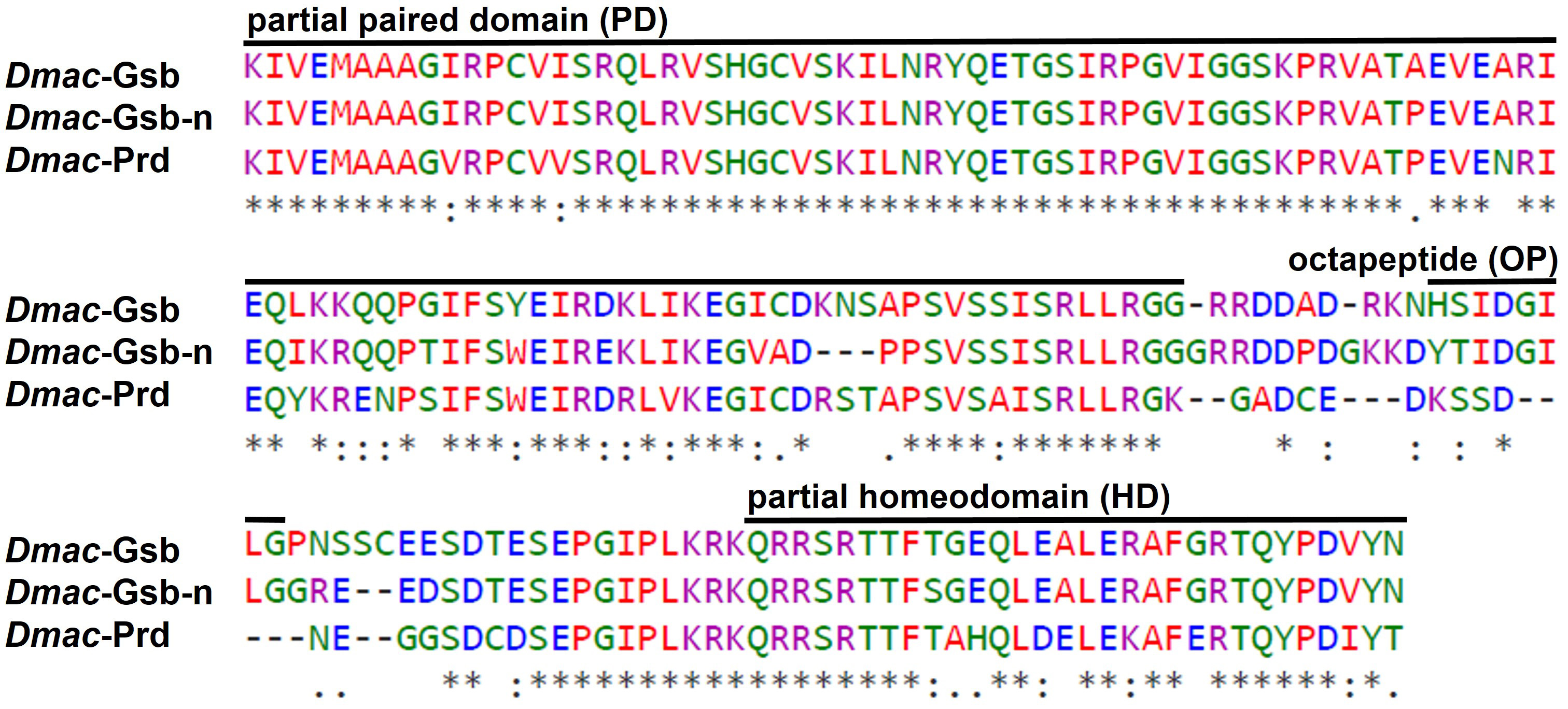

Supplement: Supplementary file 6 — 10.1186/s13227-015-0028-0 Alignment of partial Dmac-Gsb, Gsb-n, and Prd protein sequences. Black lines indicate the paired domain (PD), octapeptide (OP) and homeodomain (HD). Note that Dmac-Prd is lacking the OP motif. Gsb has a Gsb-type OP: HSIDGILG. Gsb-n has a Gsb-n type OP: YTIDGILG. Protein sequence alignment was performed using ClustalW2. * indicates identical residue, : indicates conserved substitutions, . indicates weakly similar substitutions. Colors indicate residues are classified into groups according to their physicochemical properties. Red: Nonpolar side chain; Green: Polar side chain; Blue: Negatively charged side chain; Magenta: Positively charged side chain. [file 13227_2015_28_MOESM6_ESM.tif]
