# Supplementary material for: Dermestes maculatus: an intermediate-germ beetle model system for evo-devo
Source: EvoDevo. 2015 Oct 16;6:32. doi: 10.1186/s13227-015-0028-0 (PMC4609124; doi:10.1186/s13227-015-0028-0)
Supplement: Supplementary file 2 — 10.1186/s13227-015-0028-0 Protocols for D. maculatus embryo fixation and whole mount in situ hybridization. [file 13227_2015_28_MOESM2_ESM.docx]

***D. maculatus* Embryo Fixation**

1. Prepare a collecting basket with a small piece of mesh in the center.

2. Transfer appropriately staged embryos to the basket.

3. Treat embryos with 50% bleach for four minutes. Stir occasionally.

4. Rinse with tap water three times and embryo wash buffer once (8g NaCl, 500 µl of Triton X-100 in 2L dH_2_O). Make sure to wash all embryos off the wall of the vial to the center of the mesh.

5. Transfer the mesh to a 1.5 ml Eppendorf tube with 1 ml of dH_2_O using forcep. (Note: use ~ 200 µl of embryos in each tube. If there are too many embryos, split them to more tubes.)

6. Remove the mesh once most embryos are off the mesh.

7. Submerge the tube with embryos in boiling water for 3 minutes.

8. Transfer the tube to ice immediately and let it stay on ice for 7 minutes.

9. Remove dH_2_O without losing embryos.

10. Add 500 µl of heptane and 500 µl of 4% PFA (paraformaldehyde).

11. Fix the embryos on the shaker at high speed (250 rpm) for 20 minutes.

12. Remove the aqueous phase (PFA, lower layer) without sucking up embryos.

13. Add 800 µl of 100% MeOH. Cap the tube and shake it vigorously for 20 seconds.

14. Flick the tube gently to let the embryos sink to the bottom. Note: If most embryos are still floating around, shake the tube for another 20 seconds.

15. Remove as much solution as possible without losing any embryos.

16. Rinse twice with 800 µl of 100% MeOH. Make sure to rinse embryos off the wall of the vial.

17. Add 800 µl of 100% MeOH and store the tube at -20° C.

The embryos will be dissected in PBST to remove the eggshell before in situ hybridization or antibody staining.

**Whole mount *D. macµlatus* embryo in situ hybridization with digoxigenin-labeled probe**

Rinse means invert tube several times. Wash/rock means leave tube on nutator for certain time period.

**Day 1**

1. Remove 1.5 ml Eppendorf microcentrifuge tube from freezer. Each tube should contain ~ 200 µl embryos.

2. Remove MeOH, and rinse once with 500 µl of MeOH.

3. Remove, rinse once with 500 µl of MeOH/PBST 1:1.

4. Remove, rinse three times with 500 µl of PBST.

5. Transfer embryos to a multi-well glass plate using a P1000 pipette tip with the end cut off.

6. To remove eggshell, hand-dissect embryos in PBST using Dumont #5 forceps.

7. Transfer embryos back to a 1.5 ml Eppendorf microcentrifuge tube.

8. After all the embryos sink to the bottom, remove as much PBST as possible without losing any embryos.

9. Rinse twice with PBST.

10. Remove, add 500 µl of 4% PFA, Rock for 25 min.

11. Remove PFA, rinse once with 500 µl of PBST.

12. Remove, wash three times with PBST (5 min each). * At this time turn on heat block, set the temperature to 95° C.

13. Remove most PBST but keep embryos immersed in PBST. Heat the microcentrifuge tube with embryos in heat block for 5 min (95° C). *After this, switch the heat block temperature setting to 90° C.

14. Rinse once with 500 µl of Hybridization solution (Hyb. Sol.)/PBST 1:1.

15. Remove, rinse once with 500 µl of Hyb. Sol.

16. Remove, add Hyb. Sol. 500 µl and incubate in 60° C hybridization oven for 30 min.

17. Repeat Step 16.

18. Heat the probe (1 to 50 dilution in Hyb. Sol., 200 µl in total) at 90° C in heat block for 5 min. Transfer it to ice immediately.

19. Take the microcentrifuge tube out of the oven. Remove as much Hyb. Sol. as possible without losing any embryos.

20. Add the probe. Flick the tube gently a few times and incubate it at 60° C overnight (~ 16 hours).

**Day 2**

1. Carefully remove probe. Rinse once with 500 µl of Hyb. Sol.

2. Remove, add 1 ml of Hyb. Sol., rock for 20 min at 60° C.

3. Remove, add 1 ml of Hyb. Sol./PBST 1:1, rock for 20 min at 60° C.

4. Remove, wash 4X5 min in PBST at 60° C.

5. Remove, add 1 ml of 1:2000 anti-dig-AP, FAB-fragment antibody (diluted in PBST).

6. Incubate for 1 hour at room temperature (R.T.).

The following steps are all carried out at R. T.

7. Remove, rinse once with 500 µl of PBST.

8. Remove, wash 4X10 min with PBST.

9. Remove, wash in 500 µl of staining buffer for 5 min.

10. Remove, add 1 ml color reaction buffer (4.5 µl of NBT 100 mg/ml and 3.5 µl of BCIP 50 mg/ml in 1 ml of staining buffer).

11. Rock in dark, check color change every 10 min in multi-well glass dish under microscope.

12. Rock until color develops to ideal intensity. Stop reaction by adding 500 µl of PBST.

13. Remove solution. Rinse three times with PBST.

14. Wash with PBST 3X5 min.

15. Wash with MeOH/PBST 1:1 for 5 min.

16. Rinse twice with MeOH.

17. Rinse once with EtOH.

18. Rinse twice with MeOH.

19. Wash with MeOH/PBST 1:1 for 5 min.

20. Wash with PBST for 3X5 min. * Embryos can be saved in PBST at 4° C for days before visualization.

To visualize embryos:

Embryos at pre-blastoderm, blastoderm and gastrulation stages can be visualized directly in PBST under dissecting microscope.

Germ band needs to be dissected out from surrounding yolk with forceps. To mount, germ band is transferred onto microscope slide with either a P200 tip (end cut off) or with forceps. After removing the remaining PBST, add 70% glycerol (in 0.1 M Tris pH 8.0) on to microscope slide and flatten germ band carefully with forceps. Cover with a coverslip and then proceed to visualization using microscope.

**20XSSC (1L)**

175.3 g NaCl

88.2 g Sodium citrate

adjust pH to 7.0

store at R.T.

**Hybridization Solution (50 ml)**

50% Formanmide 25 ml

5XSSC 12.5 ml of 20X

100 ug/ml Salmon Sperm DNA 500 µl

50 ug/ml Heparin 250 µl

0.1% Tween 20 50 µl

dH_2_O 11.7 ml

store at 4° C

**Staining Buffer (50 ml)**

100 mM NaCl 1 ml of 5M

50 mM MgCl_2_ 2.5 ml of 1 M

100 mM Tris pH 9.5 46.45 ml

0.1% Tween 20 50 µl

store at R.T.

**Troubleshooting and comments on protocol**

1. If a lot of embryos at the blastoderm stage are not intact or many germ bands have been fragmented after fixation, hand shaking might be too vigorously. Try to reduce shaking time or shake less vigorously.

2. Poor fixation might result if there are too many embryos in each individual Eppendorf tube. Try to split embryos into more tubes.

3. Fixation still works without the heating and cooling treatments. But the space between the eggshell and the embryo might be small for hand-dissection if you skip this step.

4. We have tried several ways to get around the requirement for hand-dissection to remove the eggshell but they were unsuccessful. These included extending bleach treatment time and using ice cold MeOH during fixation.

5. For in situ hybridization, the color starts to show up after ~ 15 minutes and develops within an hour. a) If the staining becomes dark very quickly or shows strong background, try to use more diluted probe. b) If the staining is still weak after one hour, remove solution and add fresh NBT/BCIP solution. Also, using less diluted probe might help.
