# Supplementary material for: Dermestes maculatus: an intermediate-germ beetle model system for evo-devo
Source: EvoDevo. 2015 Oct 16;6:32. doi: 10.1186/s13227-015-0028-0 (PMC4609124; doi:10.1186/s13227-015-0028-0)
Supplement: Supplementary file 5 — 10.1186/s13227-015-0028-0 D. mauclatus early embryogenesis at 25 and 30 °C. Embryos were collected every 2 h AEL at 25 or 30 °C over an 18-h or a 10-h period, respectively. D. maculatus embryogenesis was examined using nuclear and phalloidin staining. Embryos at the end of 8–10 h AEL at 30 °C are roughly equivalent to 14–16 h AEL embryos at 25 °C. [file 13227_2015_28_MOESM5_ESM.pdf]

	25 °C	30 °C
**0-2 hr AEL**	Male and female pronuclei fuse	Male and female pronuclei fuse, zygotic nuclei divide in the center of the egg
**2-4 hr AEL**	Zygotic nuclei divide in the center	Most nuclei migrate toward the egg periphery, a syncytial blastoderm forms
**4-6 hr AEL**	Zygotic nuclei divide and gradually distribute along the embryo	Cellular blastoderm forms, gastrulation starts
**6-8 hr AEL**	Most nuclei migrate toward the egg periphery, a syncytial blastoderm forms	Gastrulation completes, germband extention begins
**8-10 hr AEL**	Cellular membrane form between individual energids, a cellular blastoderm forms, gastrulation starts	Serosal window closes, germband extends dorsally
**10-12 hr AEL**	Gastrulation proceeds, an early germ band with an open serosal window is established	
**12-14 hr AEL**	Germ band extends, head segmental furrows become visible	
**14-16 hr AEL**	Germ band extends dorsally, thoracic segmental furrows become visible	
**16- 18 hr AEL**	Germ band is fully extended, abdominal segments become obvious, appendage buds form	
